# Response of Degarelix treatment in human prostate cancer monitored by HR-MAS ^1^H NMR spectroscopy

**DOI:** 10.1007/s11306-016-1055-0

**Published:** 2016-06-30

**Authors:** Basetti Madhu, Greg L. Shaw, Anne Y. Warren, David E. Neal, John R. Griffiths

**Affiliations:** Cancer Research UK Cambridge Institute, Li Ka Shing Centre, University of Cambridge, Robinson Way, Cambridge, CB2 0RE UK; Department of Urology, Cambridge University Hospitals NHS Trust, Cambridge, UK; University College London Hospitals NHS Foundation Trust, London, UK; Department of Pathology, Cambridge University Hospitals NHS Trust, Cambridge, UK; Nuffield Department of Surgery, John Radcliffe Hospital, University of Oxford, Headington, Oxford, UK

**Keywords:** Prostate, Cancer, Metabolomics, Degarelix, Metabolism, NMR, HR-MAS

## Abstract

**Introduction:**

The androgen receptor (AR) is the master regulator of prostate cancer cell metabolism. Degarelix is a novel gonadotrophin-releasing hormone blocker, used to decrease serum androgen levels in order to treat advanced human prostate cancer. Little is known of the rapid metabolic response of the human prostate cancer tissue samples to the decreased androgen levels.

**Objectives:**

To investigate the metabolic responses in benign and cancerous tissue samples from patients after treatment with Degarelix by using HRMAS ^1^H NMR spectroscopy.

**Methods:**

Using non-destructive HR-MAS ^1^H NMR spectroscopy we analysed the metabolic changes induced by decreased AR signalling in human prostate cancer tissue samples. Absolute concentrations of the metabolites alanine, lactate, glutamine, glutamate, citrate, choline compounds [t-choline = choline + phosphocholine (PC) + glycerophosphocholine (GPC)], creatine compounds [t-creatine = creatine (Cr) + phosphocreatine (PCr)], taurine, myo-inositol and polyamines were measured in benign prostate tissue samples (n = 10), in prostate cancer specimens from untreated patients (n = 7) and prostate cancer specimens from patients treated with Degarelix (n = 6).

**Results:**

Lactate, alanine and t-choline concentrations were significantly elevated in high-grade prostate cancer samples when compared to benign samples in untreated patients. Decreased androgen levels resulted in significant decreases of lactate and t-choline concentrations in human prostate cancer biopsies.

**Conclusions:**

The reduced concentrations of lactate and t-choline metabolites due to Degarelix could in principle be monitored by in vivo ^1^H MRS, which suggests that it would be possible to monitor the effects of physical or chemical castration in patients by that non-invasive method.

**Electronic supplementary material:**

The online version of this article (doi:10.1007/s11306-016-1055-0) contains supplementary material, which is available to authorized users.

## Introduction

Prostate cancer is the second commonest cause of cancer death in males in the UK (Thompson et al. 2007). Nuclear magnetic resonance (NMR) spectroscopy methods have been used, both in vivo and ex vivo, to differentiate between benign prostate hypertrophy (BPH) and malignant prostate cancer samples (Fowler et al. 1992). A unique metabolic trait of the prostate gland is abnormal citrate metabolism: normal prostate accumulates high citrate concentrations and secretes it into the seminal fluid (Costello et al. 1999). This metabolic trait is lost in prostate cancer. In addition, the multiply-coupled methylene protons of three polyamines (putrescine, spermidine (a dimer of putrescine) and spermine (a dimer of spermidine) overlap; this results in broad ^1^H NMR resonances between the choline and creatine signals that are detectable by NMR in the normal prostate and are reduced in cancer. Liu et al. found increased gene expression and activity of ornithine decarboxylase (ODC), a rate limiting enzyme in the polyamine biosynthesis pathway that synthesizes putrescine from ornithine, in benign prostatic hyperplasia samples (Liu et al. 2000). Spermine, a polyamine that is found to be reduced in prostate cancer tissue samples when measured by high-resolution magic angle spinning (HRMAS) ^1^H NMR, showed a linear correlation with the volume percentage of normal epithelial cells that were quantified by histopathology methods (Cheng et al. 2001). Additionally, ^1^H NMR spectroscopic studies on malignantly transformed human prostatic cells often show increased choline compounds, suggesting altered membrane phospholipid metabolism (Ellen Ackerstaff et al. 2001). Importantly, Santos et al. (2010) have shown that HRMAS can be performed on human tissue without losing transcriptomic data. They concluded that despite a decrease in RNA integrity number (RIN) following HRMAS spectroscopy, analysis of expression microarrays demonstrated no significant change in gene expressions between HRMAS-analysed and control samples of surgical and biopsy tissues.

^1^H NMR measurements of citrate from human seminal fluid and expressed prostatic fluid have been shown to outperform serum PSA in identifying prostate cancer (Kline et al. 2006), and citrate, myo-inositol and spermine have also been shown to be potential age-independent biomarkers of prostate cancer in human expressed prostatic secretions (Serkova et al. 2008). Tessem et al. investigated the use of lactate and alanine as metabolic biomarkers of prostate cancer by using HRMAS ^1^H NMR spectroscopy on biopsy samples (Tessem et al. 2008). An analysis of the metabolite profiles of human prostate tissue extracts, obtained from HR-NMR spectroscopy concluded that citrate and spermine decreased whereas lactate and choline increased in prostate cancer samples (Kumar et al. 2014). In another recent study, HR-NMR data analysis of serum samples from prostate cancer patients and healthy controls established that the biomarkers alanine, pyruvate and glycine distinguished 90 % of cancer cases (84.4 % sensitivity and 92.9 % specificity) and also discriminated 92.9 % of low grade cases (92.5 % sensitivity and 93.3 % specificity) (Kumar et al. 2015). All these above-mentioned studies were aimed at establishing magnetic resonance methods either to distinguish normal prostate from benign (and also from malignant) neoplastic tissue or to follow the metabolic changes in comparison with the progression of indices of malignancy and/or aggressiveness (factors such as cellularity and Gleason grade) of the prostate cancer. These studies have quantified the metabolites either in relation to an internal standard like trimethylsilyl propanoic acid (TSP) (Swanson et al. 2008, 2006) or by using the external electronic signal reference method known as electronic reference to access in vivo concentrations (ERETIC) (Albers et al. 2009). The use of relative metabolite concentrations or metabolite ratios may lead to ambiguous results if the supposedly constant metabolite in the ratio actually changes, so several methods have recently been proposed for obtaining absolute concentrations (Albers et al. 2009; Ratiney et al. 2010). LCModel, which has been widely used for estimating metabolite concentrations from in vivo ^1^H MRS data of brain, has also been used on HRMAS ^1^H NMR data from human brain tumours tissue samples (Piccirillo et al. 2012) and prostate cancer biopsies (Garcia-Martin et al. 2011; Madhu et al. 2014; DiCamillo et al. 2005). We have developed a modified LC-model basis set in order to get absolute concentrations of metabolites from human prostate biopsies.

Androgen deprivation therapy (ADT) is the standard clinical approach to the management of advanced prostate cancer; nowadays drugs are used to lower the serum androgen levels (this was historically achieved by surgical removal of the testes) resulting in clinical remission in 95 % of cases (Bruchovsky et al. 2006). A recent phase III trial concluded that Degarelix (a novel gonadotropin-releasing hormone antagonist) achieved rapid suppression of PSA levels and castrate testosterone levels irrespective of baseline levels in prostate cancer patients (Damber et al. 2012). In another study, Degarelix showed a rapid reduction of testosterone levels in prostate cancer patients, with 95 % of patients having castrate levels of serum testosterone 28 days after administration (Klotz et al. 2008). A recent study showed that Degarelix inhibits prostate cell growth in normal, hyperplastic and cancer cell lines, possibly by involvement of a cell-cycle related mechanism, leading to apoptosis (Sakai et al. 2015) and another recent study has evaluated the effects of Degarelix treatment on human prostate cancer by immunohistochemistry and transcript profiling (Shaw et al. 2015). However there is no data available showing how the metabolism of human prostate tissue is affected by Degarelix treatment. In the present study, the HRMAS ^1^H NMR spectroscopic method was used to analyse surgically resected human benign and prostate cancer samples with and without Degarelix treatment (tissue removed surgically 7 days after administration of Degarelix from patients with confirmed castrate levels of serum testosterone) in order to evaluate the metabolic changes associated with medical castration. To the best of our knowledge this is the first study to investigate metabolic changes due to Degarelix treatment in prostate cancer tissue samples.

## Materials and methods

### Clinical Sample collection

Full ethical approval was obtained for all elements of the study including clinical sample collection and analysis: NCT01852864 for Degarelix-treated patients (REC ref: 11/H0311/2) and NCT00967889 for untreated patients (REC ref: 01/4/061).

The study was designed to examine the early effects of chemical castration (by Degarelix administration) on human prostate cancer. Patients were recruited to the study through the urology outpatient clinic at Cambridge University NHS Trust, Cambridge, UK. Six patients with high-risk prostate cancer (PSA > 20 ng/ml or Gleason grade > 7 or clinical stage ≥ cT2c) were administered 240 mg of Degarelix S.C. (donated by Ferring Pharmaceuticals) in Addenbrooke’s Clinical Research Facility, 7 days before undergoing radical prostatectomy.

These patients were matched for known risk factors (age, serum PSA, tumour grade and stage) with ten patients who underwent radical prostatectomy without neoadjuvant Degarelix. For all patients the prostate was sampled and samples snap frozen as described (Warren et al. 2013). We took care to minimise the time that the tissue samples were left at room temperature as the exposure of surgical samples to room temperature can increase the lactate levels. Samples obtained from patients who had not received Degarelix are referred to as untreated. Prostate samples found to contain cancer are called untreated cancer; prostate samples where no cancer was found are called untreated benign. For patients administered Degarelix, serum samples were taken immediately before administration of Degarelix and immediately prior to surgery for measuring pre- and post-Degarelix treatment testosterone levels respectively. Serum testosterone levels at the time of surgery were confirmed as castrate for all patients treated 7 days previously with Degarelix (data not shown). See Table 1 for patient and tumour characteristics.

**Table 1:** Baseline characteristics of degarelix-treated and untreated cohorts

		Degarelix-treated cancer (n = 6)	Untreated cancer (n = 7)	Untreated benign (n = 10)
Age (years) [median(range)]		60.52 (47–68)	60.42 (51–67)	61.43 (51–68)
PSA (ng/ml) [median(range)]		8.59 (5.5–12.2)	10.32 (5.8–18)	11.51 (5.2–13.9)
Clinical stage	1	1	2	2
	2	2	3	7
	3	3	2	1
Biopsy Gleason score	6	1	1	1
	7	3	5	7
	8	1	0	1
	9	0	1	1
	10	1	0	0
Pathological stage at prostatectomy	2	1	3	3
	3	5	4	7
Prostatectomy Gleason score*	7	4	6	8
	8	0	0	1
	9	2	1	1
Nodal status	negative	4	6	0
	positive	2	1	1

* Post-prostatectomy Gleason score must be interpreted with caution as prostate cancer architecture and therefore Gleason score is affected by hormonal therapy, even if short-lived. Mild architectural changes were observed in the post-treatment group. No patients had received any hormonal treatment with a 5-alpha reductase inhibitor other than Degarelix within 6 months prior to undergoing radical prostatectomy

Tissue cores were obtained using a published method (Warren et al. 2013). Slices from each end of the cores were stained with H&E and examined by a specialist uropathologist (AW), and the presence or absence of tumour was confirmed. Untreated means the patients had not been exposed to presurgical Degarelix. Twenty-three post-surgical prostate samples were snap frozen in cryogenic vials (in liquid nitrogen), and preserved at −80 °C until the NMR analysis. Ten prostates were sampled from patients who had not been exposed to Degarelix. Samples containing cancer were isolated from seven of these prostates, (untreated cancer) and samples which did not contain prostate cancer (untreated benign) were obtained from all ten of the prostates. Samples containing prostate cancer were obtained from the prostates of six patients who had been treated with Degarelix (treated cancer).

One tissue sample was analysed at a time by transferring it on dry ice from the −80 °C freezer to a Category 2 containment cabinet. The sample was cut to size on the dry ice so as to fit into the 4 mm plastic Bruker HRMAS rotor insert. The sample weight was determined by weighing the insert before and after loading with the sample (weights for samples; benign prostate = 14.63 ± 1.55 mg, untreated cancer = 8.13 ± 0.58 mg, Degarelix-treated cancer = 7.18 ± 1.55 mg). A small screw was fitted onto the top of the insert and the whole capped insert was inserted into the Bruker 4 mm HRMAS NMR zirconium rotor. The top screw of the rotor was fitted and the rotor was placed into the NMR spectrometer.

### Metabolite data acquisition

HRMAS ^1^H NMR data acquisition was performed on a Bruker 600 MHz instrument with a 4 mm HRMAS probe. All the spectra were obtained using TOPSPIN 2.5 Bruker software and at a spin rate of 3000 Hz and a sample temperature of 4 °C. A water-suppressed pulse sequence with a repetition time of 8 s, 128 transients and 64 K time domain points was used to get the metabolite spectrum. The corresponding water spectrum was acquired with 8 s repetition time, eight transients and 64 K time domain points. A water-suppressed CPMG pulse sequence with acquisition parameters of 8 s repetition time, 128 transients and 64 K time domain points was used with a T_2_ filter (T_2_ filter times with 50 ms, 100 ms and 200 ms) to acquire metabolite spectra with suppression of the broad lipid and macromolecule signals. The total data acquisition time for each sample was about 1 h 30 min.

### Data analysis

#### Multivariate analysis

We analysed the metabolite profiles from prostate tissue samples by using the pattern recognitions methods principal component analysis (PCA) and orthogonal projections to latent structures-discriminant analysis (OPLS-DA). The spectra were binned from 0.5 to 4.5 ppm with 0.01 ppm intervals. Binned data were exported to SIMCA (Umetrics^®^) software for multivariate analysis. All the bins were mean-centred and scaled (with Pareto scaling) and PCA analysis was conducted to find out the general trends and outliers in the data, while OPLS-DA was used to get a clear discrimination between the groups. Scores plots were used for classification of the samples and loadings plots to identify the metabolites responsible for the separation of samples in the scores plots.

#### Metabolite quantitation

LCModel software (http://www.s-provencher.com/pages/lcmodel.shtml) was used on water-suppressed spectra to estimate the metabolite concentration (Provencher 1993). A modified LCModel basis set was used. Since these were not brain tumours, NAA and NAAG were omitted from the analysis; instead, citrate, polyamines and phosphocreatine (PCr) signals were simulated. Crammer–Rao lower bound values (standard deviation of 20 %) in LCModel were used to estimate reliable fittings of metabolites from NMR spectra. The absolute metabolite concentrations were quantified relative to the water signal observed in each individual experiment (Madhu et al. 2014; Madhu et al. 2006; Piccirillo et al. 2012) and then Student’s *t* test (two tailed) was carried out. The methodology for estimation of metabolite concentrations was validated with phantoms containing known concentrations of metabolites. The dye used in the process of punching the biopsy gets mixed with the tissue sample and it produces a single peak at about 3.70 ppm in the HRMAS ^1^H NMR spectrum. We also simulated this signal so as to get an optimum residual between the observed and fitted spectrum in the LCModel fittings. Supplementary Table 1s shows the metabolites with their ChEBI identifiers and abbreviations used in this study.

## Results

**Fig. 1:**
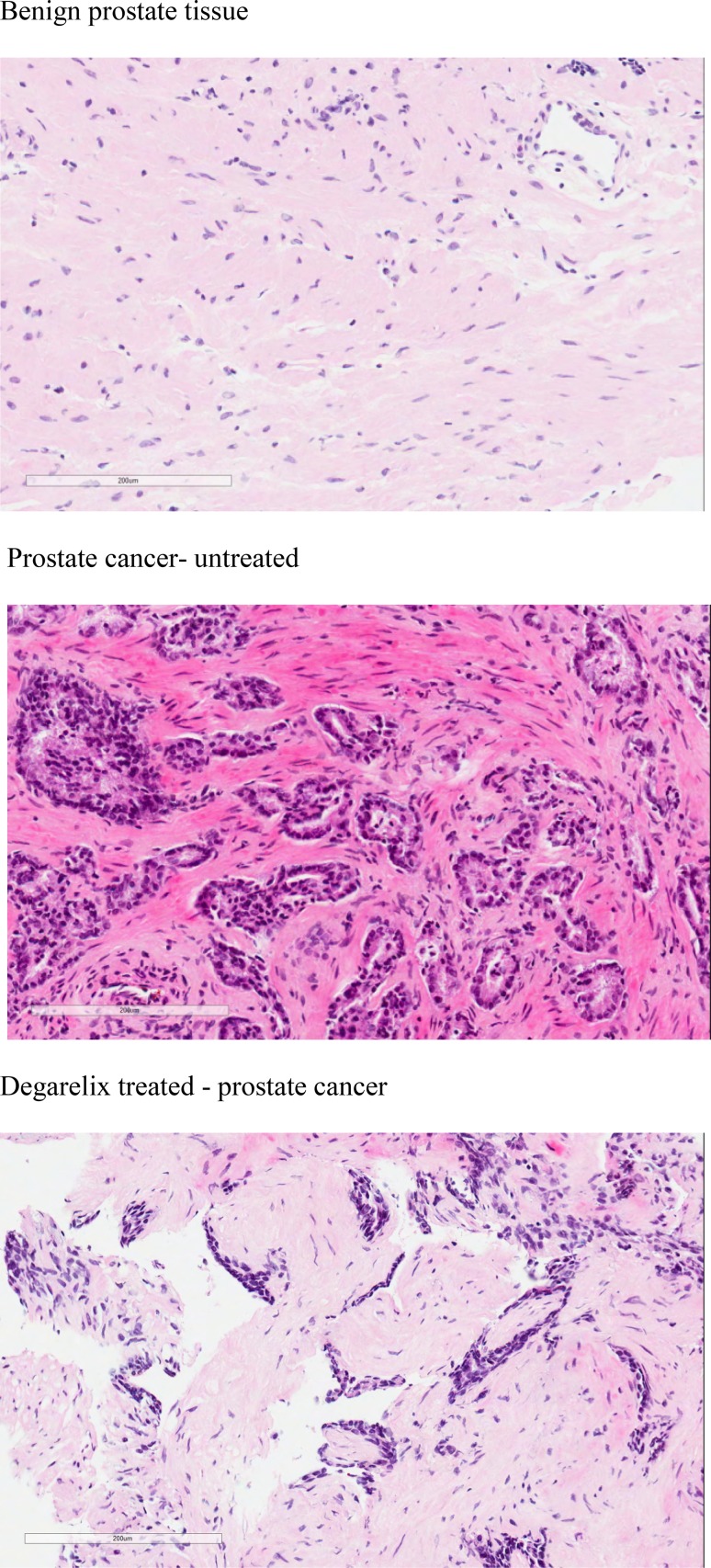
H&E sections from the patient prostate tissue samples

Table 1 shows the characteristics of the patients included in this study and their prostate cancers. Median age, biopsy grade, pathological stage and nodal status of the samples were not significantly different between untreated (control) and Degarelix-treated groups. Histology has been the “gold standard” for evaluating cellular malignancy in routine clinical diagnosis for prostate cancer and Fig. 1 clearly shows the cellular differences between human benign prostate and both untreated and treated prostate cancer tissues. Degarelix-treated histological sections show a degree of hormonal change, with more vacuolation and apoptosis compared to the untreated prostate cancer samples.

**Fig. 2:**
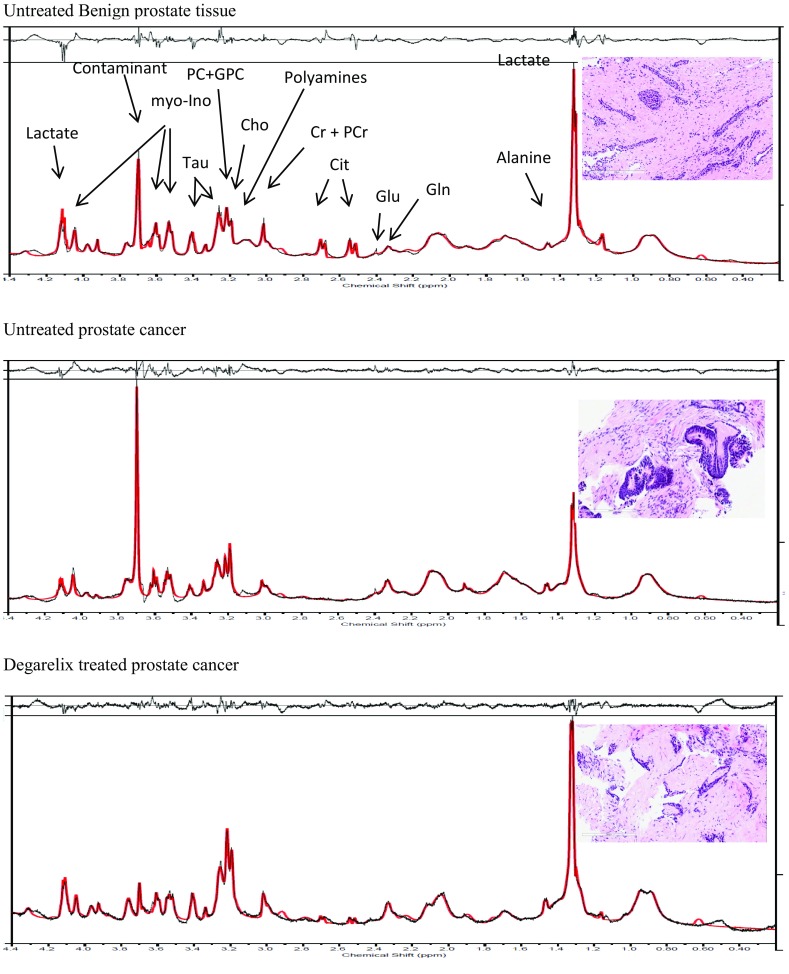
LC Model fittings of water suppressed HRMAS ^1^H NMR spectra from prostate tissue samples. Residuals between the observed and fitted spectrum are also shown on each of the spectra. (*Gln* glutamine *Glu*-Glutamate, *Cit* citrate, *Cr* creatine, *PCr* phosphocreatine, *Cho* choline, *PC* phosphocholine, *GPC* glycerophosphocholine, *Tau* taurine, *myo-Ino* myo-Inositol)

Figure 2 shows the observed HRMAS ^1^H NMR spectra (in black) from benign, untreated cancer and Degarelix-treated human prostate tissue samples; the LCModel fitted spectrum line appears in red. The residuals, the difference between the observed (black) and fitted spectrum (red) appear on the top of the plots, showing that these spectral fittings were optimal. The metabolite concentrations estimated from LCModel fittings to HRMAS ^1^H NMR spectra of human prostate samples are presented in Table 2.

**Table 2:** Estimated concentrations (in mM) of metabolites, lipids and macromolecules obtained from LCModel fittings of HRMAS ^1^H NMR spectra of prostate samples

Metabolites	Untreated-benign prostate tissue	Untreated prostate cancer tissue	Degarelix treated prostate cancer tissue
	mean ± SEM	mean ± SEM	mean ± SEM
Alanine	1.13 ± 0.24	2.48 ± 0.18	2.09 ± 0.23
Acetate	3.04 ± 1.07	5.32 ± 1.58	3.19 ± 1.13
Choline (Cho)	1.62 ± 0.40	2.95 ± 0.73	1.43 ± 0.32
Creatine (Cr)	1.73 ± 0.29	1.59 ± 0.35	2.02 ± 0.43
Glutamine (Glu)	7.43 ± 2.74	13.90 ± 5.61	3.79 ± 1.66
Glutamate (Gln)	5.51 ± 1.32	11.20 ± 3.06	5.12 ± 0.72
Glycine	1.25 ± 0.18	1.56 ± 0.00	1.95 ± 0.27
PCh + GPC	1.74 ± 0.21	3.32 ± 0.66	2.81 ± 0.28
Myo-inositol	9.17 ± 1.84	10.75 ± 2.10	7.79 ± 1.04
Lactate	15.59 ± 1.90	23.31 ± 2.54	15.21 ± 1.89
Lysine	4.44 ± 1.68	8.32 ± 3.25	2.20 ± 0.52
Phosphoethanolamine (PE)	15.38 ± 6.44	32.70 ± 13.35	8.99 ± 5.35
Scyllo-Inositol	2.34 ± 0.86	3.94 ± 1.37	1.34 ± 0.32
Taurine	6.40 ± 1.19	7.76 ± 1.29	6.02 ± 0.43
Phospho-Creatine (PCr)	1.63 ± 0.48	2.15 ± 0.81	1.05 ± 0.07
Citrate	5.82 ± 2.00	2.20 ± 1.05	3.05 ± 0.63
t-choline (Cho + GPC + PCh)	3.14 ± 0.38	5.66 ± 0.68	3.48 ± 0.51
t-creatine (Cr + PCr)	2.59 ± 0.36	3.06 ± 0.52	2.15 ± 0.32
Glu + Gln	12.66 ± 3.91	24.59 ± 8.31	8.62 ± 1.34
Lipids and macromolecules (MM)			
Lipid 0.9 ppm	28.92 ± 3.93	31.48 ± 7.61	16.10 ± 4.34
MM 0.9 ppm	66.48 ± 0.00	10.56 ± 0.00	13.61 ± 1.51
Lipid 2.0 ppm	164.55 ± 101.44	206.02 ± 101.84	21.11 ± 12.04
MM 2.0 ppm	72.99 ± 18.43	114.85 ± 40.39	59.05 ± 22.04
Lip 1.5 ppm	1420.01 ± 229.64	1781.03 ± 682.35	544.69 ± 367.80
MM 1.4 ppm	5.50 ± 1.88	9.98 ± 2.03	9.84 ± 4.19
MM 1.7 ppm	243.13 ± 129.43	226.36 ± 165.19	60.00 ± 35.75
Polyamines	162.53 ± 65.74	191.87 ± 87.74	50.96 ± 9.56
Lipid 1.3 ppm	42.70 ± 6.77	55.42 ± 13.08	33.10 ± 8.77

*SEM* standard error mean, *PCh* phosphocholine, *GPC* glycerophosphocholine

**Fig. 3:**
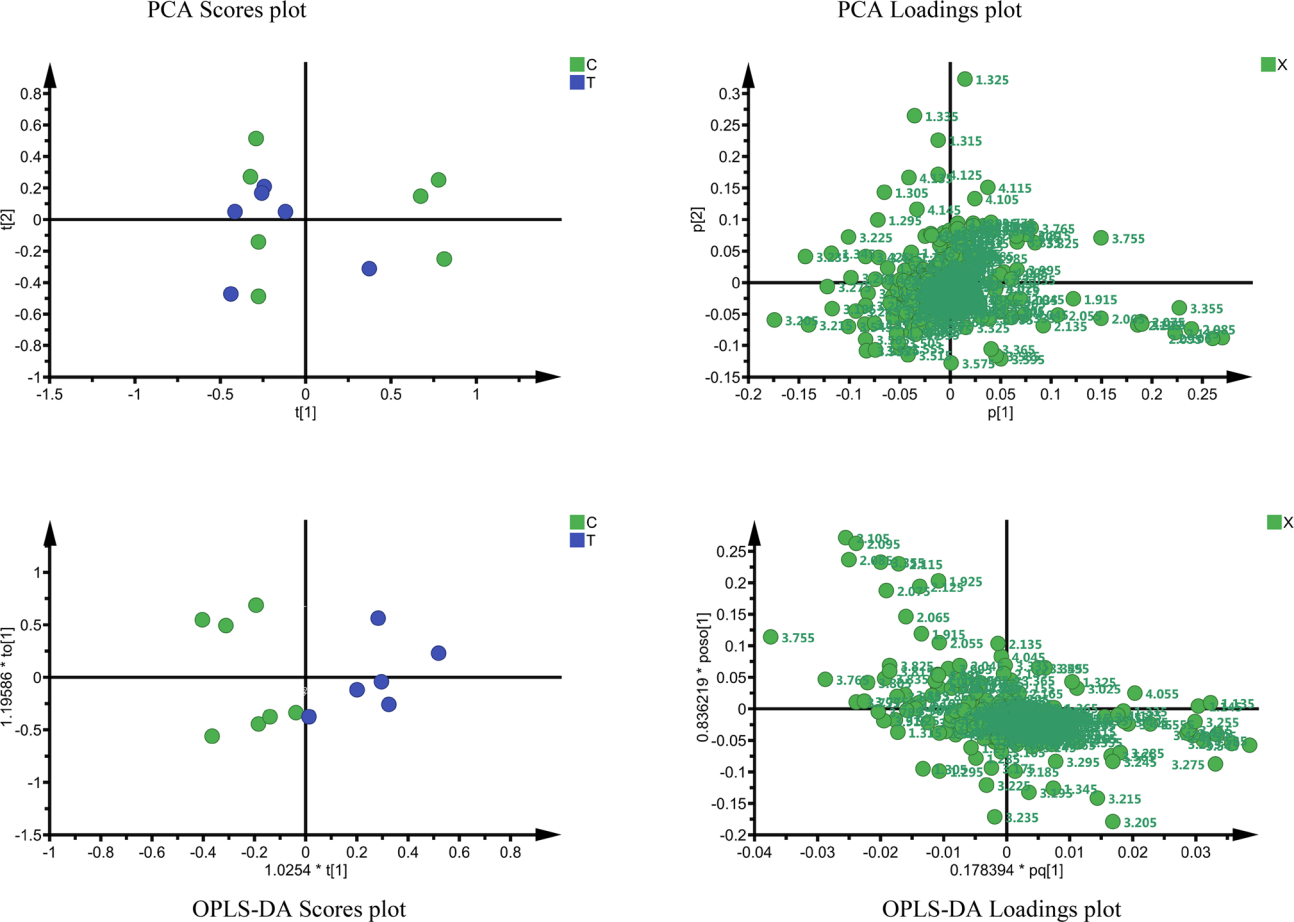
Results from Principal Component Analysis (*top left* and *right panels*) and OPLS-DA (*bottom left* and *right panels*). *Green dots* on the *left panels* are samples from untreated patients (*C*) and *blue dots* are samples from Degarelix-treated patients (*T*)

The PCA scores plots showed a trend of separation of untreated and Degarelix treated prostate cancer samples, as can be seen in Fig. 3 (top panel), but it could not completely separate the two groups. We therefore performed OPLS-DA (Fig. 3 bottom panel) in an attempt to obtain a complete discrimination. The scores plot of OPLS-DA (Fig. 3 bottom left panel) shows a clear separation of the untreated and treated groups (though one sample from each group overlaps). Information about the chemical shifts can be obtained from the OPLS-DA loadings plot (Fig. 3 bottom right panel) which shows that the bins containing signals from metabolites such as choline-containing compounds, lactate, glutamine, glutamate and myo-inositol are causing the separation of two groups that had been observed in the OPLS-DA scores plot.

**Fig. 4:**
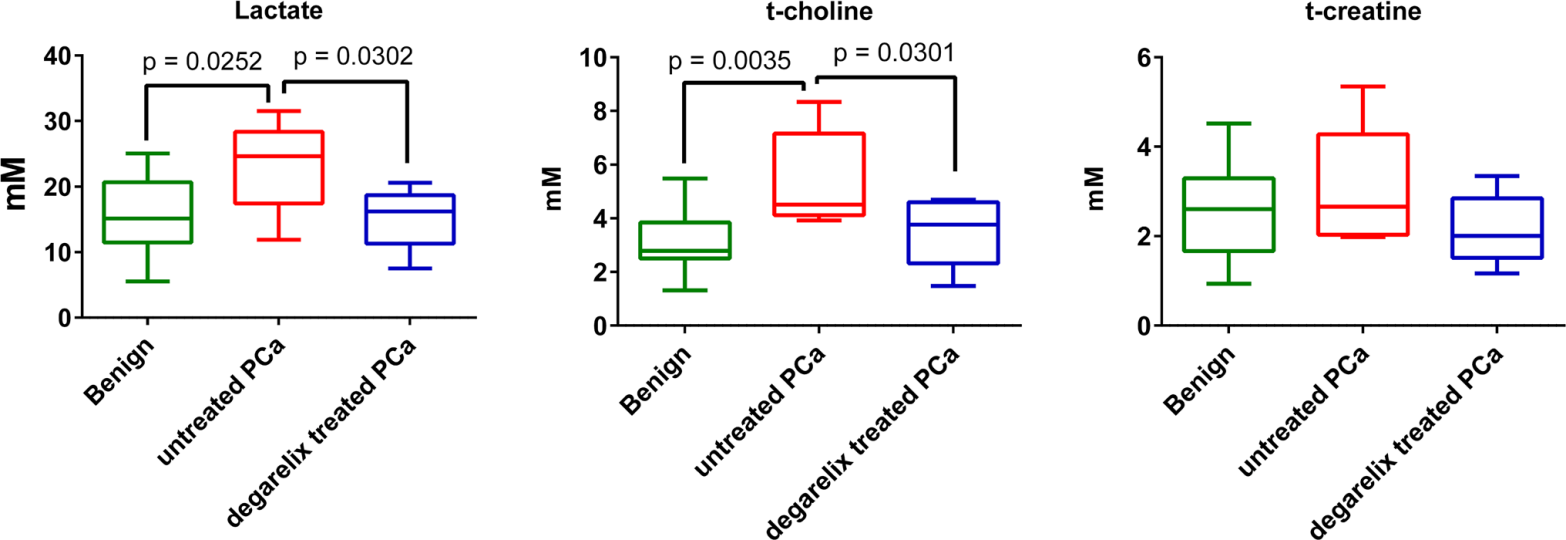
Lactate, t-choline and t-creatine metabolite changes in benign, untreated and Degarelix-treated prostate cancer

Figure 4 shows that lactate and t-choline [choline + phosphocholine (PC) + glycerophosphocholine (GPC)] concentrations were significantly higher in the prostate cancer samples compared to benign samples. The t-creatine [creatine (Cr) + phosphocreatine (PCr)] levels did not show any significant differences in cancer samples compared to benign samples (Fig. 4; Table 2). Citrate signals could not be found in four out of seven cancer samples. Lactate and t-choline concentrations in Degarelix-treated cancer samples were significantly lower than in untreated samples, while citrate and t-creatine levels did not show any significant differences (Table 2). Though there was a general tendency towards lower levels of alanine, choline, glutamate, taurine and myo-inositol in prostate samples from Degarelix-treated patients, the differences were not statistically significant (Table 2). In general the ranges and quartiles of the Degarelix treated samples were smaller compared to both benign and untreated prostate cancer samples (Table 2; Fig. 4; supplementary Fig. 1s).

In contrast, the benign tissue did not show any significant changes following Degarelix treatment in any of the metabolites estimated in this study (data not shown). Many previous studies that are reviewed in the introduction and discussion sections used metabolite ratios as markers to discriminate the normal from the malignant samples, and such data have been shown to be correlated with Gleason grade (van Asten et al. 2008). In this study, however, we found that metabolite ratios such as t-choline/t-creatine, t-choline/citrate, (t-choline + t-creatine)/citrate and (t-choline +t-creatine + polyamines)/citrate were not different between the Degarelix-treated and untreated prostate cancer samples (supplementary figure S2).

## Discussion

Histopathological examination is the gold standard for differentiating benign from cancerous prostate tissue in clinical practice. In vivo and ex vivo NMR methods have also been used to successfully distinguish the metabolic differences between normal, benign and malignant tissues, and these studies have generated new insights into treatment-induced remodelling of metabolism of the tumour tissues. However, there is a paucity of data regarding the metabolic effects of androgen deprivation on human prostate cancer. We have used samples from a clinical study of Degarelix to track the rapid metabolic changes taking place in response to androgen deprivation. HRMAS ^1^H NMR represents a unique non-destructive method by which to evaluate the metabolic changes to follow the anti-cancer treatment.

### Comparison of benign and tumour metabolites

The elevated lactate and alanine levels observed in tumour samples compared to benign samples indicate enhanced glycolysis (Fig. 4; Table 2). These samples showed the well-known metabolic phenotype called the “Warburg effect”, which has been found in many cancer cells and malignant tumour tissues (DeBerardinis et al. 2008; Warburg et al. 1927; Ward and Thompson 2012). However, one has to be careful with interpretation of lactate concentrations in the ex vivo tissue samples as the exposure of surgical samples to the room temperature can increase the lactate levels.

Increased levels of choline and choline-containing compounds have frequently been observed in cancer, by both in vivo and by ex vivo MRS studies on cancer biopsies and cell extracts (Bertilsson et al. 2011, 2012; Giskeødegård et al. 2013; Selnaes et al. 2013). Similarly, the t-choline and (PC + GPC) contents observed the prostate cancer samples were significantly higher than in benign prostate.

The higher glutamine and glutamate levels in cancer biopsies compared to untreated benign prostate tissue samples might be due to increased glutaminolysis (DeBerardinis et al. 2007). The citrate levels in normal prostate were found to be in the range of 5 mM–13 mM depending on central or peripheral zone of prostate tissue, whereas the citrate concentration in prostate cancer tissue was about 1–3 mM (Costello et al. 1999; Costello et al. 2002). In the case of malignant prostate tissue citrate values can be less than 0.5 mM (Costello et al. 1999). We could not observe citrate signals in four out of seven prostate cancer tissue samples. Our estimated citrate levels in normal and prostate cancer tissue samples (Table 2) are in the same range as these above mentioned literature values.

### Degarelix effect on tumour metabolites

Reduced lactate levels in Degarelix-treated prostate cancer might be an indication of lower glycolysis (Table 2; Fig. 4). Alanine, another product of glycolysis, showed a similar trend but it did not reach statistical significance (Table 2). A small increase of citrate levels was found in Degarelix-treated prostate cancer compared to untreated cancer samples, but it was not statistically significant (Table 2). Total choline levels were significantly reduced after Degarelix treatment (Table 2; Fig. 4). These lower t-choline levels indicate that Degarelix has an effect on membrane phospholipid metabolism. In a recent study choline kinase alpha (CHKA) expression was decreased in tissues from patients treated with Degarelix, both at the transcript and protein levels, compared with control patients without androgen deprivation (Asim et al. 2016). Gene expression data from these samples (Shaw et al. 2015) shows a general trend of decrease of expression of protein encoding genes along the major metabolic pathways. Degarelix treatment did not change the glutamine and glutamate levels significantly (Table 2; supplementary Fig. 1). In general, the plots of metabolite concentrations show that the levels of metabolites in the samples from the patients treated with Degarelix have smaller ranges compared to samples from untreated patients. The fact that even a study of this size, where very small numbers of samples are involved, could find significant changes in the lactate and t-choline metabolite concentrations shows the potential of using HRMAS ^1^H NMR spectroscopy as a method to study the metabolic effects of androgen deprivation therapy. Moreover these metabolites can be readily observed on clinical MRI scanners (even at 1.5T field strength), which makes them potential biomarkers for following androgen deprivation therapy by non-invasive in vivo ^1^H MRS. In clinical applications, observation of the lactate signal might require spectral editing, since some of the lipid signals observed by in vivo ^1^H MRS of human prostate overlap with lactate.

### Could the effects of castration be reflected in metabolite ratios?

HRMAS ^1^H NMR data from the prostate are sometimes expressed as metabolite ratios with respect to citrate (Kobus et al. 2015). The concentrations of spermine, citrate and the ratio CCP/C [(total choline + creatine + polyamines)/citrate] obtained from HRMAS ^1^H NMR of human prostate tissues samples data using the peak assignment and quantification program LCModel (Linear Combination Model) showed that the cancer tissues could be distinguished from normal tissue with a sensitivity of 86.9 % and specificity of 85.2 %. (Giskeødegård et al. 2013). Metabolite ratios estimated from the HRMAS ^1^H NMR data of human prostate needle biopsies have shown a positive correlation of choline/creatine (also t-choline/creatine) with Gleason score while a negative correlation was observed for citrate/creatine with Gleason score (van Asten et al. 2008). These ratios are not always reliable indicators of progression or response, as citrate levels are very low or even undetectable by NMR in prostate cancer tissue samples. The metabolite ratio of [(PC) + (GPC)]/creatine has been found to be correlated with the proportion of cancerous tissue in human prostate samples (Stenman et al. 2011). In our study, metabolite ratios normalised to t-creatine levels did not change significantly after Degarelix treatment whereas the absolute concentration of t-choline was significantly lower. Even though membrane blebbing is a well-known feature of apoptosis the choline levels do not change markedly, whereas creatine levels decrease, possibly due to the cellular disintegration process (Madhu et al. 2006). In this present study the failure of the t-choline/t-creatine ratio to change significantly may have been due to a simultaneous decrease in both metabolite concentrations (Madhu et al. 2006) since the absolute concentrations show significant differences. Thus metabolite ratios estimated through MR methods may not be reliable indicators for following removal of testosterone in prostate cancer.

## Conclusions

Absolute concentrations of alanine, lactate, glutamine, glutamate, citrate, t-choline, t-creatine, taurine, myo-inositol and polyamine metabolites were measured in samples of benign and (untreated and Degarelix treated) prostate cancer tissue from patients. Lactate, alanine, t-choline and (PC + GPC) concentrations were significantly elevated in high-grade prostate cancer tissue when compared to benign prostate tissue samples. Degarelix treatment resulted in significant decreases of lactate and t-choline concentrations in the prostate cancer samples.

Lactate and t-choline metabolite signals from the prostate gland in humans can be monitored non-invasively by in vivo ^1^H MRS on standard clinical MRI scanners. The reduced concentrations of lactate and t-choline due to Degarelix treatment that have been observed in this study indicate that there is a potential application of in vivo ^1^H MRS for monitoring androgen deprivation therapy non-invasively during human prostate cancer treatment. It may also be possible in the future to use MRS to localise prostate cancer clones that are resistant to the metabolic effects of androgen deprivation and subject them to targeted treatment.

## Electronic supplementary material

Below is the link to the electronic supplementary material.

Supplementary material 1 (DOCX 174 kb)
