# Supplementary material for: Response of Degarelix treatment in human prostate cancer monitored by HR-MAS 1H NMR spectroscopy
Source: Metabolomics. 2016 Jun 30;12:120. doi: 10.1007/s11306-016-1055-0 (PMC4927592; doi:10.1007/s11306-016-1055-0)
Supplement: Supplementary file 1 — Supplementary material 1 (DOCX 174 kb) [file 11306_2016_1055_MOESM1_ESM.docx]

**Supplementary figures**


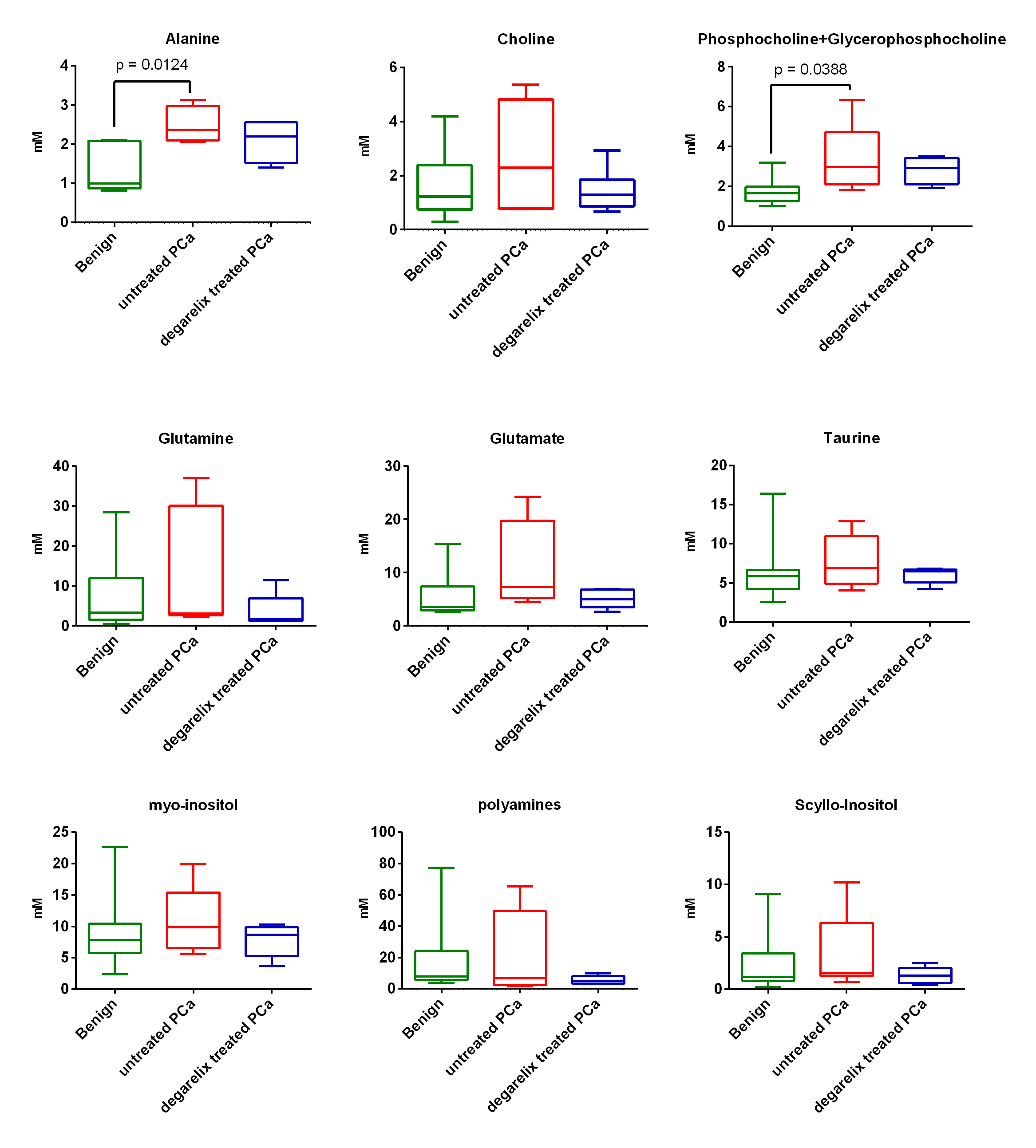


Supplementary Figure S1. Metabolite changes in benign, untreated and degarelix treated patient prostate cancer biopsies.


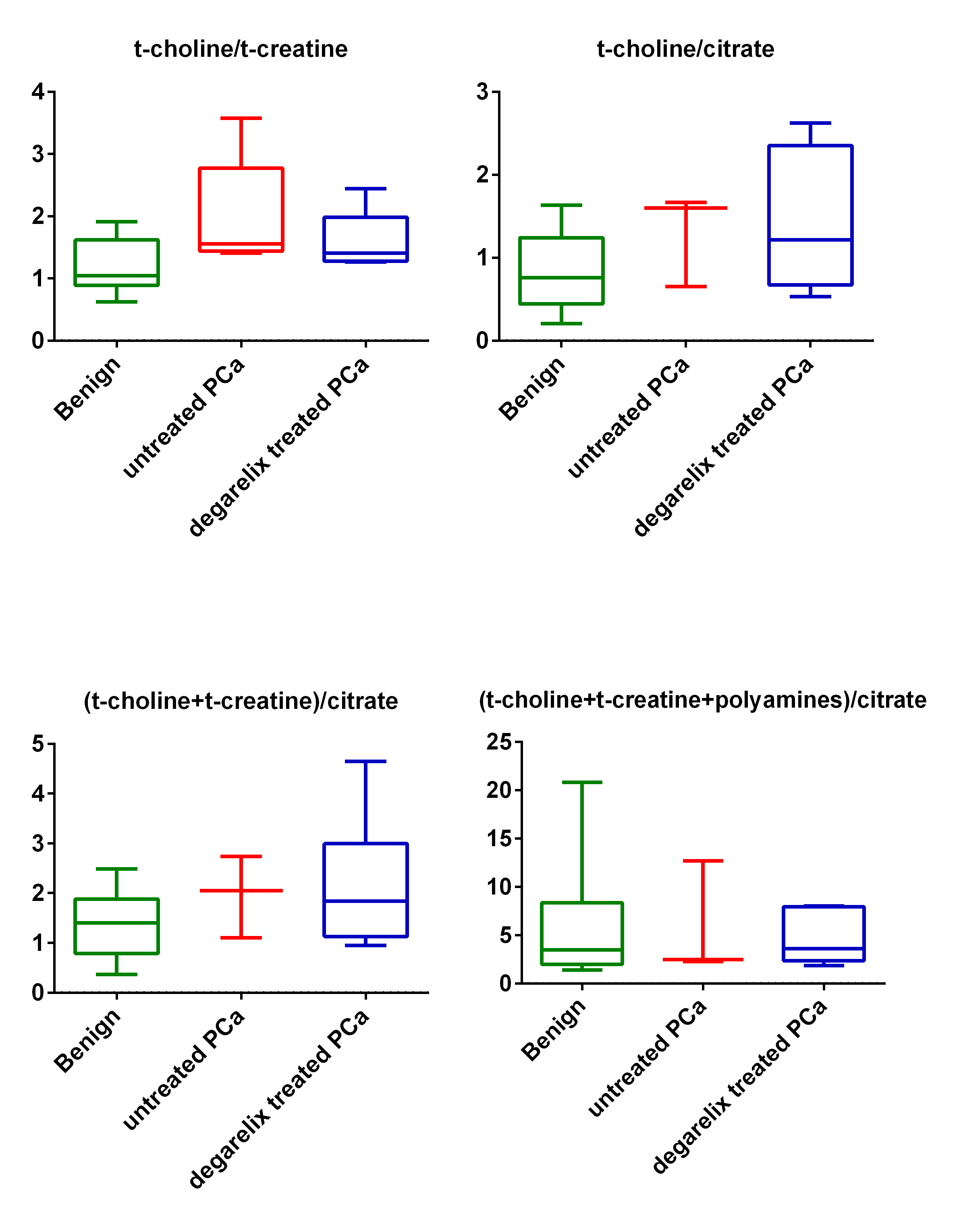


Supplementary Figure S2. Metabolite ratios normalized to t-creatine and citrate in benign, untreated and degarelix treated patient prostate cancer biopsies.

Table 1s Metabolites in the study with their corresponding CHEBI identifiers

| **Metabolite**  **(CHENOMX software)** | **CHEBI Identifier** | **Abbreviated in manuscript** |
| --- | --- | --- |
|  |  |  |
| **Lactate** | CHEBI:24996 | **Lactate** |
| **L-Alanine** | CHEBI:16977 | **Alanine** |
| **Pyruvate** | CHEBI:15361 | **pyr** |
| **Creatine** | CHEBI:16919 | **Cr** |
| **N-Phosphocreatine** | CHEBI:17287 | **PCr** |
| **Choline** | CHEBI:15354 | **Cho** |
| **O−Phosphocholine** | CHEBI:18132 | **PC** |
| **O−Phosphoethanolamine** | CHEBI:17553 | **PE** |
| **sn−Glycero−3−phosphocholine** | **CHEBI:36313** | **GPC** |
| **Glutamate** | CHEBI:18237 | **Glu** |
| **Glutamine** | CHEBI:28300 | **Gln** |
| **Citrate** | CHEBI:30769 | **Cit** |
| **Glycine** | CHEBI:15428 | **gly** |
| **Taurine** | CHEBI:15891 | **Tau** |
| **myo−Inositol** | CHEBI:17268 | **myo−Ino** |
